# NETosis as an oncologic therapeutic target: a mini review

**DOI:** 10.3389/fimmu.2023.1170603

**Published:** 2023-04-18

**Authors:** Sarah Jaboury, Kenny Wang, Kim Maree O’Sullivan, Joshua Daniel Ooi, Gwo Yaw Ho

**Affiliations:** ^1^ Department of Oncology, Monash Health, Clayton, VIC, Australia; ^2^ School of Clinical Sciences, Monash University, Melbourne, VIC, Australia

**Keywords:** neutrophil, NETosis, cancer, innate immunity, tumor microenvironment

## Abstract

Neutrophil Extracellular Traps (NETs) are a key form of pro-inflammatory cell death of neutrophils characterized by the extrusion of extracellular webs of DNA containing bactericidal killing enzymes. NETosis is heavily implicated as a key driver of host damage in autoimmune diseases where injurious release of proinflammatory enzymes damage surrounding tissue and releases 70 known autoantigens. Recent evidence shows that both neutrophils and NETosis have a role to play in carcinogenesis, both indirectly through triggering DNA damage through inflammation, and directly contributing to a pro-tumorigenic tumor microenvironment. In this mini-review, we summarize the current knowledge of the various mechanisms of interaction and influence between neutrophils, with particular attention to NETosis, and cancer cells. We will also highlight the potential avenues thus far explored where we can intercept these processes, with the aim of identifying promising prospective targets in cancer treatment to be explored in further studies.

## 1 Introduction – the innate immune system and cancer

Cancer immunotherapy is centered on the host immune system eliminating cancer by recognizing these cells as foreign. The human immune system consists of two key components, adaptive and innate immune systems, that work closely together in a highly complementary manner to protect the host against a plethora of invading pathogens (1). In order to do so, the immune system needs to first be able to identify these pathogens and the infected cells as well as having the ability to distinguish between self from non-self molecules, which are also known as antigens (1). The adaptive immune system’s role in cancer immunosurveillance is well established and plays a crucial role for most cancer immunotherapies such as immune checkpoint inhibitors for example anti-PD1 (pembrolizumab and nivolumab) and CTLA-4 inhibitors (ipilimumab) (2, 3). The role of innate immunity in cancer surveillance is still being investigated.

The innate immune system is the first line of defense against most pathogens and is designed to rapidly react to invading foreign organisms (1). Most innate immune cells are phagocytes including neutrophils, macrophages, and monocytes (4). Phagocytes are derived from myeloid progenitor stem cells, which digest invading pathogens by phagocytosis. Tissue resident macrophages and mast cells are the first responders to tissue insults. These cells are able to excrete various cytokines and chemokines to initiate acute inflammation where other innate cells are drawn to the infected (or insulted) tissue (5). Failure of the initial acute inflammatory response to eliminate the invading pathogens often lead to activation of other immune cells including T cells, which may result in the involvement of the adaptive immune system. Most acute inflammatory responses resolute with tissue repair phase with the monocytes play an role in initiating the tissue repair processes. Chronic inflammation can occur in some circumstances when there is a failure to eliminate the offending pathogens leading to the development of a pro-tumorigenic microenvironment within the tissue.

This article explores the role of neutrophils, which are key immune cells in the innate immune system, with particular attention to the neutrophilic function of “NETosis” in promoting cancer growth and metastasis. We also explore potential avenues to target these that could be used as or in tandem with immunotherapies to improve outcomes for cancer patients.

## 2 The role of NETosis as a key function of neutrophils in innate immunity

Neutrophils comprise 50-70% of leukocytes within the blood stream (4). They are one of the first immune cell responders during the initial acute inflammatory phase to infection, environmental exposure, and cancer development (5). When activated, neutrophils have three major mechanisms of destroying pathogens and abnormal cells: phagocytosis, secretion of cytotoxic enzymes (degranulation), and release of neutrophil extracellular traps (NETs) by NETosis. NETs are an extra-cellular network of decondensed chromatin, which form weblike DNA structures which contain cytosolic and granule proteins (6). These structures are coated with various proteins within the neutrophilic nucleus, including cytotoxic enzymes used in degranulation and phagocytosis such as neutrophil elastase (NE) and myeloperoxidase, histones, and cytosolic proteins (A8, A9, A12, actin, and alpha-actinin) (7). Invading pathogens are entrapped within NETs, where they are then proteolyzed by the phagocytes.

NETosis is activated through a wide variety of mechanisms include increase in intracellular calcium concentration through β2 integrin, presence of reactive oxidative species (ROS), and activation of various signaling cascades, such as the Raf-MEK-ERK-MAP kinase pathway and SYK-PI3K-mTorc2 pathways (8, 9). They can also be activated by plasma membrane surface receptors, such as toll like receptor (TLR) 1, CD18, nucleotide oligomerization domain (NOD)-like receptor protein 2 and phorbol myristate acetate (PMA), presence of bacterial toxins, or cytokines such as complement 2 or interleukin (IL) 1 (6, 7). The activation of rapidly inducing NETosis can be either NADPH oxidase (NOX) or mitochondrial reactive species dependent (10, 11). Protein kinase C (PKC) can also phosphorylate NOX to produce ROS which then triggers NETosis.

The NOX dependent NETosis is triggered in distinct steps. It begins with the migration of MPO and Neutrophil elastase (NE) to the nuclear envelope. The NE then partially degrades the histones thus promoting chromatin decondensation. Simultaneously, hypercitrullination of histones is mediated by protein-arginine deiminase type 4 (PAD4), which is a nuclear enzyme that citrullinates arginine residues by converting the amine groups to ketones (12). PAD4 can also mediates chromatin decondensation, which is the defining feature of cellular rearrangement in the formation of the NET (13). This process is followed by actin cytoskeletal disassembly and extracellular release of decondensed DNA by exocytosis or during plasma membrane lysis (mediated by NE and the pore forming GASDERMIN D), depending on the type of NETosis (13).

**Figure 1:**
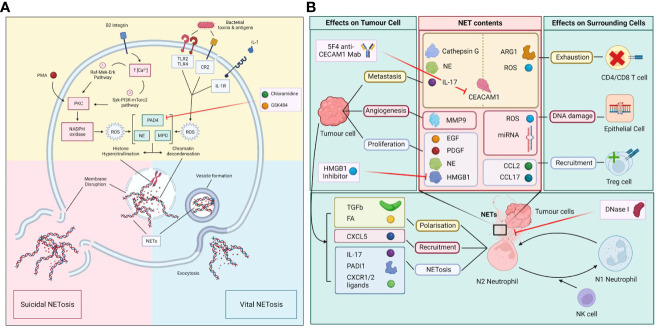
**(A)** Molecular process of vital and suicidal NETosis. The two forms of NETosis include suicidal NETosis and vital NETosis. Suicidal NETosis involves the dissolution of the plasma membrane while vital NETosis preserves the membrane with exodosis of NETs via vesicles. The two pathways converge upon the activation of PAD4, NE and MPO which leads to histone hypercitrullination, chromatin decondensation, the formation of NETs and the disruption of nuclear membrane. **(B)** Polarization of N1/N2 neutrophils in the tumour microenvironment (TME) and the effect of NETs on tumour and surrounding cells. Neutrophils in the TME are polarized towards either N1 or N2 phenotypes, which is determined by the influence of factors produced by surmunding NK cells and tumour cells. N2 neutrophils that undergo NETosis release a myriad of molecules and enzymes that have immunosuppressive and pro-tumour properties as well as causing damage to surrounding epithelial cells. In turn tumour cells release various factors that induce the polarization, recruitment, and trigger NETosis in N2 neutrophils.

There are two types of NETosis: the suicidal NETosis and the vital NETosis (**Figure 1A**). Suicidal NETosis is driven by the activation of NOX-dependent pathways through increase in intracellular calcium, which lead to the generation of ROS (7). This in turn activates myeloperoxidase (MPO), which triggers neutrophil death through rupture of the nuclear membrane to release NET. Vital NETosis, in contrast, is a NOX-independent process and is triggered by stimulation of neutrophil surface TLRs by complement proteins or microorganisms. Vital NETosis does not result in the lysis of nuclear or plasma membrane, but involves the extrinsic release of vesicles from an intact neutrophil that does not undergo cell death and continues to perform other cellular functions. Both suicidal and vital NETosis are mediated by PAD4, as described above. Also, both MPO and NE aid in histone citrullination and DNA decondensation for both types of NETosis (14).

## 3 The role of NETosis in carcinogenesis and immunotherapy resistance

### 3.1 NETosis as a driver of chronic inflammation and carcinogenesis

NETosis can play a direct and indirect role in carcinogenesis. Chronic inflammation is a well-established precursor to carcinogenesis (15). Pathological inflammation damages host tissues and induces a hyperproliferative state, which predisposes to increased risk of somatic mutations and subsequent cancer development. Whilst neutrophils appropriately activate the acute inflammatory process to eliminate invading pathogens, they can also instigate chronic, pathological inflammation in diseases such as autoimmune conditions, post viral pneumonitis (such as COVID-19), and type 2 diabetes mellitus, with NETosis being a key driver (16, 17).Constant tissue damage and subsequent DNA damage by ROS release and NETosis have been implicated in the initiation of cancers. These were observed in ulcerative colitis, where activated neutrophils were found to induce G2/M checkpoint arrest and replication errors in the colonic epithelial cells leading to colorectal cancer (18).

NETs can directly promote cancer growth by induction of cellular proliferation and mediation of angiogenesis. In pancreatic and lung cancers, NETs were found to contain the matrix metallopeptidase 9 (MMP-9), a known accelerator of angiogenesis through the degradation of extracellular matrix by activated neutrophils which triggers vascular endothelial growth factor (VEGF) release (19).

NETs can also promote cancer cell metastasis. Increased interleukin-17/Granulocyte colony-stimulating factor (IL17/GCSF) axes, a known inducer of NETosis, triggered by increased neutrophil levels have been associated with metastasis of lung and breast cancers (20–22). B2-integrin (CD18), a key cell surface protein in the activation of NETosis, has been positively correlated with metastases of colorectal cancer to the liver in mice (23, 24). In non-alcoholic steatohepatitis, NETosis has been linked to hepatocellular cancer development and metastasis (25, 26). Neutrophil activation by high mobility group box 1 protein (HMGB1) is implicated in melanoma metastasis (27, 28).

Autophagy, a lysosome-dependent degradation process of intracellular components, plays an important role in NETosis. ROS, one of the triggers of NETosis, can both induce and inhibit autophagic signaling (29). In phorbol myristate acetate (PMA)-stimulated neutrophils, autophagy and ROS production were required for NET formation (30). In acute promyelocytic leukemia cells, autophagy was found to induce NETosis (31). Autophagy in NETosis is relatively understudied.

### 3.2 NETosis is an inducer of the pro-tumor N2 neutrophil phenotype

There are two neutrophil phenotypes, N1 and N2. N1 is an anti-tumor phenotype and N2 is a pro-tumor neutrophil, also known as tumor associated neutrophils (TAN). N1s are associated with high levels of TNF-alpha, CCL2 and ICAM-1, and low levels of arginase. N2s are associated with increased levels of chemokine ligand 2 (CCL2), chemokine ligand 3 (CCL3), chemokine ligand 4 (CCL4), chemokine ligand 8 (CCL8), chemokine ligand 12 (CCL12), chemokine ligand 17 (CCL17), chemokine ligand 1 (CXCL1), chemokine ligand 2 (CXCL2), interleukin 8 (IL-8) and chemokine ligand 16 (CXCL16). N1s exhibit enhanced NOX activity, which directly triggers tumor cell death through ROS release. N1s can indirectly mediate cancer cell cytotoxicity through the activation of T and B lymphocytes, NK cells, and dendritic cells. In contrast, N2s trigger NETosis, which leads to the development of an unfavorable tumor microenvironment and the release of pro-angiogenic factors (**Figure 1B**) (32). Interestingly, NETosis can generate hypercoagulative states and induction of N2 phenotype transformation in small intestinal neoplasia in mice (33). Whilst more N1s can be detected in the early stages of carcinogenesis, a switch from N1 to N2 phenotype is seen at later stages of cancer progression, indicating a link between the tumor microenvironment and neutrophils to trigger a neutrophil metabolic reprogramming to a pro-tumor phenotype (18). TANs have been shown to upregulate fatty acid transport protein 2, mediated by STAT3 and STAT 5, leading to increased cancer cell uptake of exogenous fatty acids and can induce reprogramming of N1s to N2s (18). In murine mammary tumors, IL-1β release by TANs during NETosis can upregulate IL-17 expression that can cause gCSF dependent TAN recruitment (34–36). IL-1β can promote pancreatic cancer development in a process involving crosstalk between TANs, adipocytes and pancreatic stellate cells (37). TANs have higher NADPH oxidase and ROS activities compared to N1 cells, which are associated with increased inflammation and inhibition of T cell function (18).

### 3.3 NETosis can generate an unfavorable tumor microenvironment

The tumor microenvironment includes the constitution of connective tissue, blood vessels, extracellular matrix, and inflammatory cells that infiltrate and reside in the tumor stroma. Within the tumor microenvironment, NETosis can promote cancer growth and metastasis. NETs can physically trap circulating cancer cells whilst cathepsin G (neutrophil derived serine protease) can cause the release of insulin like growth factor 1. The insulin like growth factor 1 can increase E cadherin-mediated intercellular adhesion and promote cancer cell aggregation and entry into blood vessels (38, 39).

NETs can also induce proliferation of cancer cells through NE and HMGB1 (13). In *in vitro* models of triple negative human breast cancer and murine lung cancer, NET formation stimulated the invasion and migration of the tumor cells (40). Carcinoembryonic antigen-related cell adhesion molecule 1 (CEACAM1) is particularly tropic to neutrophils and found abundantly in NETs. CEACAM1 blockade, through 5F4 (CEACAM1 monoclonal antibody) or NE inhibitor Sivelestat, can significantly decrease colonic carcinoma migration, suggesting that CEACAM1 is key to cancer cell metastasis (41). NET-DNA scaffold recognition by Coiled-Coil Domain Containing 25 (CCDC25) in cancer cells can activate the ILK-β-parvin pathway to promote cancer cell proliferation and migration (42).

Epithelial to mesenchymal transition (EMT) is one of the fundamental processes in cancer metastasis (43). NETosis can promote EMT in breast and gastric cancers (44, 45). This process is thought to be driven by NE, which induces tumor cell migration *via* the activation of Src/PI3K/Akt pathway (46).

NETosis has been linked to chemotherapy resistance in cancers. In a mouse model of multiple myeloma, neutrophils promoted chemoresistance to doxorubicin and melphalan through soluble factors released in the tumor microenvironment during NETosis (47, 48). MMP9, released in NETs, are a key driver of angiogenesis in cancers and corelates with poorer sensitivity to oxaliplatin in gastric cancer. The inhibition of MMP-9 improved sensitivity of colorectal cancer cells to 5-FU chemotherapy (49, 50).

In cancers such as ovarian cancers that are typically immunologically cold tumors, which are associated with resistance to immunotherapies, often have immune-suppressive microenvironment (51). Recent studies showed that ovarian cancer cells can induce NETosis by neutrophils within the tumor microenvironment to create a premetastatic niche within the omentum for cancer cells to seed whilst evading immune detection (52). Endometrial cancers are also typically immunologically ‘cold’ and NETosis has been detected in these cancers on a tissue level, with circulating cell free DNA and citrullinated histone H3 are being explored as potential biomarkers of NETosis in these endometrial cancers (53).

Lastly, NETosis can be linked to resistance of cancer immunotherapies as it creates a favorable tumor microenvironment for tumor immune evasion. In pancreatic ductal adenocarcinomas (PDACs), high expression of IL-17 and PADI1 have been correlated with poorer prognosis. IL-17 and PADI1 blockade increased tumor cell sensitivity to anti-PD-1 and anti-CTLA4 immunotherapies (22). Tumor-produced chemokines, acting through CXCR1 and CXCR2 chemokine receptors, can induce NETosis and aid immune evasion of cancers by physically coating tumor cells and preventing contact with CD8 and NK cells (54). Additionally, PD-L1 has been detected on the surface of NETs formed by neutrophils isolated from colorectal cancer patients who had undergone resection of liver metastasis, and NETosis can induce CD4 and CD8 T cell exhaustion within the tumor microenvironment (55). CEACAM1 expression, a component of NET webs, is associated with T-cell exhaustion and resistance to tumor infiltrating lymphocytes immunotherapy in melanoma patients (38). T cell immunoglobulin and mucin domain 3 (TIM3) is an inhibitory molecule correlated with T cell exhaustion in cancers, and is a novel target for recent immunotherapy research (39). Lastly, higher IL-8 levels have been recently found to be an accurate prognosticator for poorer response to checkpoint blockade immunotherapies, likely due to its known sequelae, including activation of CXCR1 and CXCR2, triggering of angiogenesis, and immunosuppression through recruitment of immunosuppressive MDSC to tumor microenvironment (56).

## 4 Targeting neutrophils and NETosis – the current landscape

Numerous avenues for targeting neutrophils in cancer treatment have been explored (**Table 1**). PAD4, as highlighted previously, is an essential trigger of both vital and suicidal NETosis. When PAD4 was blocked in ovarian cancer by GSK484, a small molecule inhibitor specific for PAD4, metastasis to the omentum was significantly reduced (52). Another PAD4 inhibitor chlorine amidine, a pan-PAD inhibitor, can reduce cancer cell invasion and modified cancer cells to a less aggressive phenotype in a PDAC *in vitro* model (57).

**Table 1:** Potential anti-N2 and anti-neutrophil extracellular trap (NET) therapeutics and evidence.

Class	Mechanism	Compound	Evidence
PAD4 inhibitor	Block initiation of NETosis	Chloramidine	*In vivo* murine model of ovarian cancer (57)
		GSK484	*In vitro* human pancreatic adenocarcinoma cell lines (58)
TGF-β inhibitor	Reversal of N2 to N1 phenotype	Anti-TGF-beta (1D11)	*In vitro* human model of colorectal cancer (59)
		Anti-TGF-beta1 receptor (SB525334)	Mixed *in vivo* and *in vitro* murine models of pancreatic cancer (60)
Agonist of trained immunity	Enhance N1 anticancer activity	Beta glucan	*In vivo* murine model of melanoma (61)
CXCR signalling inhibitor	Reduce N2 recruitment	Pertussis toxin inhibiting G unit (Reparixin)	*In vitro* human, mixed cancers (lung, prostate, renal, pleural, urothelial) (62)
		SX-682	*In vivo* murine model of HNSCC (63)
HMGB inhibitor	Reduce initiation of NETosis	Glycyrrhizin	*In vivo* murine model of bladder cancer (64)
DNAse	Digestion of NETs	Pulmozyme	Murine model of multiple myeloma (65)
		DNAse I	Murine model of lung cancer (48)
			*In vitro* human breast cancer (38)
			*In vitro* human gastric cancer (55)
			*In vitro* human colorectal cancer (66)
NF-kB inhibitor	Reduce MMP-9 release in NETosis	Enalapril	*In vivo* human colorectal cancer (67)
NE inhibitor	Mitigate pro-metastatic effects of NE	Neutrophil elastase inhibitor	*In vitro* murine and human models of colorectal cancer (49)
EACAM inhibitor	Mitigate immunoregulatory effects of CEACAM	Monoclonal antibody to CEACAM1	*In vitro* murine and human models of colorectal cancer (49)
			*In vitro* murine model of colorectal cancer (68)
IL-17 inhibition	Prevent neutrophil migration and metastasis	Monoclonal antibody to IL17/IL17 receptor	*In vivo* murine model of PDAC (69)
NADPH inhibition	Inhibit suicidal NETosis	Diphenyleneiodonium chloride	*In vitro* mouse and human models (68, 70)
Inflammatory mediator	Increase intracellular cyclic AMP	Activated C protein	*In vitro* human cancer model (71)
		Prostaglandin E2	*In vitro* human cell lines and *in vivo* murine models (70g, 72)

AMP, adenosine monophosphate; CEACAM, Carcinoembryonic antigen-related cell adhesion molecule; CXCR, CXC chemokine receptor; HMGB, high mobility group box protein; HNSCC, head and neck squamous cell carcinoma; IL-17, interleukin 17; NADPH, nicotinamide adenine dinucleotide phosphate; NE, neutrophil elastase; NF-kB, nuclear factor kappa B; PAD4, protein arginine deiminiase 4; PDAC, pancreatic ductal adenocarcinoma; TGF-β, tumour growth factor beta.

Several studies have investigated targeting NE within NETs. BAY 85-8501 is an endogenous antiprotease NE inhibitor which has been shown to reduce NET formation in human neutrophils *in vitro* (73). Alvelestat (AZD9668), which inhibits NE in a dose-dependent manner, was effective in reducing inflammation in a murine model of acute lung injury (65, 67). Curcumin has also been found to target NE by decreasing NE-induced tumor cell proliferation in human lung adenocarcinoma cells *in vitro*; additionally, it has been linked to significantly reduce NETosis-driven host tissue damage in hepatic ischaemia-reperfusion injury in mice (64, 74).

Other studies have explored the potential of reversing pro-tumor N2 neutrophils back to N1 phenotype. NK cells may have a role in promoting N1 conversion. When NK cells were absent in an *ex vivo* neutrophil-NK cell-tumor cell tri-cell co-culture system, N2 neutrophilic action predominated and the N1 neutrophils was suppressed resulted in a net pro-metastatic effect (75). The same results were seen in pancreatic cancer, with subsequent improved response to immunotherapy in cancer cells post N2-to-N1 conversion (69). Non-molecular alterations to the tumor microenvironment may also play a role in delineating dominant neutrophil phenotype. Studies in mice and human models have shown that hypoxic tumor microenvironment led to less neutrophils recruitment associated with more anti-tumor activities (62).

TANs can also be targeted by blockade of fatty acid oxidation, which mitigates their immunosuppressive tendences (66). A murine study found anticancer activity of N1 neutrophils was enhanced with administration of β-glucan (76). Further potential avenues proposed to block protumor effects of TANs could be achieved by targeting the CXCL-8/CXCR-1/CXCR-2 axis, or targeting substances produced by TANS which promote tumor growth (77).

Targeting NETosis has also been explored. (**Table 1**) The TLR and CXCR signaling pathways are potential targets to reduce the pro-tumor effects of neutrophils. In PDAC, blockade of CXCR2 signaling inhibited neutrophil recruitment, reduced angiogenesis and metastasis (58, 78). Inhibition of TLR4/9-COX2 signaling can decrease NET-enabled metastatic activity (26). In mice models of liver injury, hydroxychloroquine displayed inhibition of NET formation through inhibition of TLR-9, ROS, and PAD4 (79). A recent study shows that the CXCR1 and CXCR2 pathways, which induces neutrophil chemotaxis and subsequent NETosis, can be blocked using pertussis toxin inhibiting G unit, Reparixin, which acts as an allosteric inhibitor of CXCR1/CXCR2 signaling (54). Administration of IL-8 antibodies *via* SX-682 (a small molecule inhibitor targeting CXCR1 and CXCR2) reduces the number of immunosuppressive myeloid-derived suppressor cells (MDSCs) within the tumor microenvironment and enhances the efficacy of adoptive cell therapy with NK cells (59). Tocilizumab, a monoclonal antibody against IL-6 receptor, can inhibit NET formation in cells of patients with rheumatoid arthritis (60). Both HMGB, which induces NET formation through a TLR4-dependent manner, and NETosis can be inhibited by targeting extracellular HMGB1 (through glycyrrhizin, a HMGB1 inhibitor) and NETs (through anti DNAse I), where a recent study showed delayed tumor growth post radiotherapy and improved overall survival. Additionally, there was improved radiation response and increased intratumoral CD8 T cell infiltration (80).

DNAse I is another potential agent to inhibit NETosis. In a mouse model of multiple myeloma, pulmozyme, a DNAse targeting extracellular DNA in NETosis, can reverse tumor resistance to doxycycline (48). DNAse I has successfully block NETs in lung cancer, with subsequent reduction in number of metastases in murine models (40). In humans, administration of DNAse I is linked to reduced metastasis in breast and gastric cancers (44, 45). In addition, DNAse I has been found to reverse NET-dependent T-cell exhaustion *in vitro* in human neutrophils isolated from patients with metastatic colorectal cancer (55). However, DNase I has a relatively short-half and is quickly inactivated by G actin proteins, for therapeutic window of DNase I is small. Nuclear-penetrating anti-DNA autoantibodies, such as the 3E10 derivative DX1, can interfere with the DNA damage response in NETosis and inhibits both suicidal and vital NETosis *in vivo* in both human granulocyte-like cells and murine neutrophils (81).

Enalapril, an antihypertensive medication, can re-sensitize colorectal cancer cells to 5-fluorouracil chemotherapy by inhibiting NF-kB and reducing MMP-9, a pro-angiogenic molecule released in NETosis (49). NE within NETs can be targeted with a NE inhibitor and CEACAM1 with a CEACAM1 monoclonal antibody, where both have been found to reduce metastasis in colonic cancer cells, with a synergistic effect when used in combination (41). In a murine colorectal cancer model, co-blockade of CEACAM1 and TIM3 with monoclonal antibodies enhanced intrinsic anti-tumor responses, highlighting a potential target to enhance the efficacy of immunotherapies (61). NETs can also be inhibited by activated C protein or prostaglandin E2, through an increase of intracellular cyclic AMP (82, 83). Ruboxistaurin, a protein kinase C inhibitor, can inhibit lipopolysaccharide (LPS) and PMA-induced NETs in neutrophils isolated from hospitalized patients with COVID-19 (84). IL-17 is another potential target, with recent studies displaying that IL-17 neutralization can prevent neutrophil migration and metastasis, which was linked to inhibition of tumor cell growth (21, 22). Prostaglandin E2 was found to inhibit the cAMP-PKA pathway induced by NETosis (63, 82). Lastly, NADPH can be targeted by diphenyleneiodonium chloride, which inhibits NADPH (68, 70).

Whilst a few clinical trials are currently underway to characterize NETosis in acute respiratory infection, there are currently no active trials examining NET inhibitors in cancer patients (71, 72).

## 5 Conclusion

Neutrophils have a clear role to play in the complex interactions between cancer cells and the innate immune system, and evidence highlights that NETosis can be hijacked and exploited by tumor cells to help cancers evade detection by immune cells and to enhance tumor growth, invasion, and metastasis. We have only begun to understand the intricacies of interaction between neutrophils and cancer cells, and the potential to target these pathways to improve the efficacy of cancer immunotherapies. Further, more comprehensive studies, both *in vitro* and *in vivo*, will help to further characterize these potential targets, paving the road for clinical trials utilizing these targets in immunotherapies with the aim of improving outcomes for patients with cancer. Lastly, although highly contentious, we should also be cautious of the use of granulocyte colony growth factor (gCSF) to promote neutrophil expansion post chemotherapy to reduce the risk of hospitalization, which has now been a common practice since the covid19 pandemic. The effect of neutrophilia in this instance on cancer treatment outcomes should be investigated.

## Author contributions

GH, KO’S, and JO contributed to conception and design of the manuscript. SJ and GH wrote the first draft of the manuscript. KW contributed to the figure. All authors contributed to the article and approved the submitted version.
